# Temporal Alignment of Longitudinal Microbiome Data

**DOI:** 10.3389/fmicb.2022.909313

**Published:** 2022-06-22

**Authors:** Ran Armoni, Elhanan Borenstein

**Affiliations:** ^1^Blavatnik School of Computer Science, Tel Aviv University, Tel Aviv, Israel; ^2^Department of Clinical Microbiology and Immunology, Sackler Faculty of Medicine, Tel Aviv University, Tel Aviv, Israel; ^3^Santa Fe Institute, Santa Fe, NM, United States

**Keywords:** microbiome, longitudinal analysis, temporal alignment, infant microbiome, metagenomic

## Abstract

A major challenge in working with longitudinal data when studying some temporal process is the fact that differences in pace and dynamics might overshadow similarities between processes. In the case of longitudinal microbiome data, this may hinder efforts to characterize common temporal trends across individuals or to harness temporal information to better understand the link between the microbiome and the host. One possible solution to this challenge lies in the field of “temporal alignment” – an approach for optimally aligning longitudinal samples obtained from processes that may vary in pace. In this work we investigate the use of alignment-based analysis in the microbiome domain, focusing on microbiome data from infants in their first years of life. Our analyses center around two main use-cases: First, using the overall alignment score as a measure of the similarity between microbiome developmental trajectories, and showing that this measure can capture biological differences between individuals. Second, using the specific matching obtained between pairs of samples in the alignment to highlight changes in pace and temporal dynamics, showing that it can be utilized to predict the age of infants based on their microbiome and to uncover developmental delays. Combined, our findings serve as a proof-of-concept for the use of temporal alignment as an important and beneficial tool in future longitudinal microbiome studies.

## Introduction

The human gut microbiome has a complex relationship with its host, holds vast impact on various physiologic processes such as immunity (Mazmanian et al., 2005), nutrition (Turnbaugh et al., 2006), and development (Cash et al., 2006), and plays a key role in health and disease (Dethlefsen et al., 2007). Notably, however, the composition of the microbiome is highly dynamic, both due to intrinsic natural variation and as a result of external perturbations (David et al., 2014). These dynamics are the subject of numerous recent studies, focusing on various temporal patterns including the study of temporal stability (Oh et al., 2016; Mitchell et al., 2020), stationarity (David et al., 2014; Gibbons et al., 2017), and seasonality (Runckel et al., 2011; Davenport et al., 2014).

Notably, these dynamical aspects of the microbiome are even more pronounced in infants, wherein the gut microbiome goes through a maturation process during the first few years of life before gaining an adult-like composition (Yassour et al., 2016). This maturation process could markedly impact healthy immune development and growth (Renz et al., 2011), alongside implications for health and disease later in life (Turnbaugh et al., 2006; Ivanov et al., 2009; Olszak et al., 2012; Kostic et al., 2015). Recent studies have accordingly aimed to link the taxonomic structure of the infant gut microbiome, as well as the pace of this maturation process, to various factors in the prenatal, perinatal, and postnatal life (Chong et al., 2018), including the mode of delivery (Dominguez-Bello et al., 2010; Romero et al., 2014; Chu et al., 2017; Mitchell et al., 2020), antibiotic exposure, and diet (Koenig et al., 2011; Arrieta et al., 2014; Wang et al., 2015; Bokulich et al., 2016; Yassour et al., 2016). Specifically, malnutrition can have a severe impact on the development of the microbiome, which, in turn, might play a role in the durability and outcomes of therapeutic food interventions (Subramanian et al., 2014). Interestingly, though, despite inter-personal differences in structure, dynamics, and pace, there seems to be a shared pattern of microbiome development before gaining a relatively stable, adult like composition, toward 3 years of age (Yassour et al., 2016).

This growing appreciation to the importance of understanding microbiome dynamics and of identifying, characterizing, and analyzing microbiome-based temporal processes (Björk et al., 2019), has led to a recent increase in the collection and investigation of microbiome longitudinal data, wherein multiple samples are collected from each individual over time. Notably, however, longitudinal microbiome data not only provide potential relevance and benefits, but also pose various statistical, analytical, and computational challenges. Indeed, numerous techniques for analyzing such data have been introduced in recent years. Such techniques include, for example, methods for modeling the temporal dynamics of the microbiome ecosystem (Mounier et al., 2008; Trosvik et al., 2014; Mcgeachie et al., 2016; Ridenhour et al., 2017; Shenhav et al., 2017), for uncovering novel links between the temporal behavior of the microbiome and various medical and biological conditions (Kostic et al., 2015; Chong et al., 2018; Leonard et al., 2021), and for distinguishing between biological and technical variation (Silverman et al., 2018).

**FIGURE 1:**
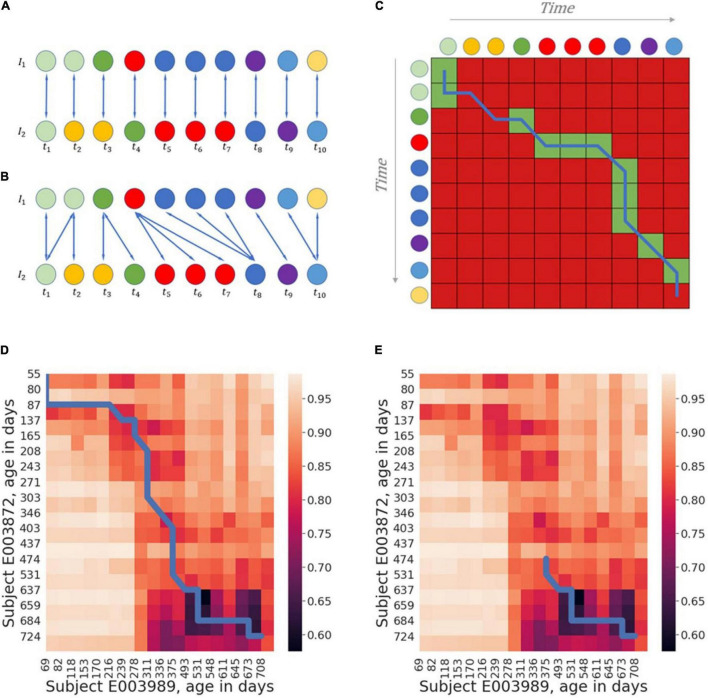
A schematic example of the temporal alignment framework. **(A)** A naïve matching of chronologically parallel samples. Top and bottom sequences of circles represent trajectories of individuals I1 and I2, with circles of the same color representing samples with similar compositions and circles of different colors represent dissimilar compositions. **(B)** A mapping that allows the matching of non-parallel samples, resulting in higher total similarity. **(C)** A heatmap based on the similarities between the samples of the two trajectories. Cells corresponding to similar samples are colored in green, while those corresponding to dissimilar samples are colored in red (for simplicity, we assume here that samples can be either similar or dissimilar whereas in reality, the measure of similarity is usually continuous). The blue line traverses the matches that were presented in panel **(B)**. **(D,E)** Example of global **(D)** and local **(E)** alignments of real microbiome data. The alignment is denoted again by the blue line, plotted on a heatmap of the pairwise Bray-Curtis dissimilarity between samples obtained from two individuals. Darker areas in the heatmap indicate more similar samples. As can be seen in this figure, the global alignment finds a path traversing the whole matrix while accumulating maximal pairwise similarity, while the local alignment only focuses on the regions with the highest similarity.

Yet, one major challenge that arises when analyzing and comparing longitudinal data is that although temporal patterns of microbiome processes (such as development) may be shared across different individuals, their pace and dynamics may differ, causing similar time-series to be “out-of-phase,” and in turn, produce low similarity values between the trajectories of these processes if not accounted for. A possible approach to address this issue is *via temporal alignment*. Temporal alignment algorithms aim to find a match between time series samples obtained from two individuals such that the overall dissimilarity between these individuals (using some dissimilarity measure) is minimized, while preserving temporal order (see Figures 1A–C). For example, Dynamic Time Warping (DTW) is a family of algorithms that carry out temporal alignment, using dynamic programming to find the optimal match between two given time-series, while considering possible compressions or stretches in time scale (Keogh and Ratanamahatana, 2005). DTW is often used in research fields involving the analysis of time series data, such as gene expression analysis (Aach and Church, 2001), speech recognition (Vintsyuk, 1968; Sakoe and Chiba, 1978), or human movement analysis (Gavrila et al., 1995), where time series often vary in pace.

Unfortunately, however, there is relatively little prior work on using alignment-based methods for temporal analysis of microbiome data. For example, aiming to cluster microbiome time-series data from infants, De Muinck and Trosvik (2018) considered the four main phyla in the relevant period of development, calculated the pairwise Euclidean dissimilarity between time-points, and then used DTW to calculate the pairwise dissimilarities between the full trajectories. Hierarchical clustering based on the mean calculated dissimilarities then successfully clustered a pair of twins that were among the 12 subjects included in this research. The “TIME” web platform (Baksi et al., 2018) also includes a built-in workflow using DTW as a distance method before the application of hierarchical clustering, but the user is given only very limited control over various aspects of the process and does not have access to the resulting alignment. Lugo-Martinez et al. (2019) used a continuous variant of temporal alignment as a preprocessing step, shifting participating trajectories according to the calculated alignment and using this alignment-based transformation for downstream analysis. These studies, while intriguing, leave major unexplored gaps in understanding the application and utility of alignment-based algorithms to microbiome data. For example, while prior work used the resulting alignment distance for downstream analysis (e.g., clustering), the alignment itself, i.e., the obtained matching between samples, likely includes rich information regarding the temporal dynamics of the participating trajectories and can be used in various settings. Furthermore, the use of the DTW algorithm was limited or anecdotal and did not explore the many settings and configurations this framework supports. And finally, such alignment-based analyses have been applied only to a couple of datasets, making it hard to draw more general conclusions about the potential benefits of this approach to microbiome research.

In this work, we therefore set out to address these gaps and to put forward a comprehensive exploration of the properties and performance of DTW-based temporal alignment algorithms in the domain of microbiome research. Since, as described above, the development of the microbiome in the first years of life is a dynamic process that poses a variety of challenges relating to longitudinal analysis, we focus on developmental data as our use case, analyzing data from 4 independent longitudinal datasets of infant microbiomes from different countries, and with various medical conditions (see Table 1). Specifically, we set out to demonstrate that alignment-based dissimilarity measures outperform simpler, more naïve measures of dissimilarity, and that the matching of samples provided by the alignment algorithm successfully facilitates age prediction and highlight developmental patterns.

**TABLE 1:** Data sources included in the analysis.

Source	# of subjects	Age range (days)	Mean # of samples per subject	Notable metadata
Bokulich et al., 2016	28	0–729	22	Mode of delivery, diet, antibiotics use.
De Muinck and Trosvik, 2018	12	1–364	222	–
Kostic et al., 2015	31	6–729	19	T1D and Seroconversion status, Mode of delivery, diet.
Subramanian et al., 2014	50 (control)	0–726	20	Diet, antibiotic use.
	23 (malnourished)	201–783	13	Malnourishment status and treatment

## Methods

### Data Acquisition and Pre-processing

Data was obtained from 4 longitudinal studies of infant microbiomes. Since the data originated from different studies and was available at different levels of processing, additional processing steps were conducted to gain consistent and comparable data. The four datasets include: (1) Bokulich et al. (2016): This study examined the effect of delivery mode and diet on the composition of the microbiome in a cohort of United States infants from birth to 2 years of age, sampled monthly during the first year of life, and every 2 months in the second year of life. The Bokulich dataset was acquired from QIITA (Gonzalez et al., 2018) under study ID 10249. (2) De Muinck and Trosvik (2018): This study sampled 12 infants from Oslo, Norway, on a near daily basis, and described temporal patterns in the acquisition of the microbiome during the first year of life. The raw sequencing reads were downloaded from the NCBI Sequence Read Archive (SRA) under accession codes: SRP141136 (ID1), SRP141176 (ID2), SRP141301 (ID3), SRP141326 (ID4), SRP141372 (ID5), SRP142048 (ID6), SRP142071 (ID7), SRP142093 (ID8), SRP142140 (ID9), SRP142235 (ID10), SRP142291 (ID11), and SRP142429 (ID12). The data was then preprocessed using QIIME2 (Bolyen et al., 2018), with DEBLUR (Amir et al., 2017) used for sequence quality control and feature table construction. (3) Kostic et al. (2015): This study explored the connection between the microbiome in infancy and T1D in a cohort of 33 infants from Finland and Estonia. The raw sequencing data was acquired from the DIABIMMUNE project website^1^, and preprocessed using QIIME2, with DADA2 (Callahan et al., 2016) used for sequence quality control and feature table construction. (4) Subramanian et al. (2014): This study compared the microbiome composition of healthy and malnourished Bangladeshi infants. A pre-processed OTU-table was downloaded from the Gordon lab’s website^2^. In this work, we used the samples originating from children with severe acute malnutrition, along with the healthy singleton and healthy twins and triplets’ cohorts. In total, these four datasets include 5,144 samples from 146 children (see Table 1). In all datasets, rare features, defined as features whose abundance wasn’t > 0.1% in at least 2 samples, were filtered out.

### Samples Interpolation

Uneven or sparse sampling can be a major challenge when analyzing longitudinal microbiome data, thus, interpolation of samples to gain denser and/or more even sampling might be beneficial for downstream analyses. In this work, we implemented a pipeline for sample interpolation using a Gaussian kernel, similar to the interpolation approach used by Alpert et al. (2018) on single-cell data, relying on the idea that an interpolated sample will likely be more similar to samples collected in time points close to it than to remote samples. Specifically, given a chronological sequence of microbiome samples and the requested sampling frequency (based either on the desired interval between consecutive samples or the desired number of samples per trajectory), the value of each feature (e.g., OTU) in each interpolated sample was calculated as a weighted sum of the values of this feature in the samples of the original trajectory, with the weights having a Gaussian distribution, each inversely related to the difference in time between the samples. Formally, the value for feature *f* in an interpolated sample at time-stamp *t* is calculated based on values *f_1_f_n_* with corresponding weights *w_1_w_n_* as:


$$f=\frac{1}{\sum_{i=1}^{n}w_{i}}\,\sum_{i=1}^{n}w_{i}\cdot f_{i}$$

where:


$$w_{i}=e^{\frac{\left(t-t_{i}\right)\,2}{w\,i\,n\,d\,o\,w^{2}}}$$

and “window” is a user-defined window size parameter used to control the level of influence of different samples in the original trajectory on the interpolated sample. In this work, unless stated otherwise, temporal interpolation was carried out with fixed 30-day intervals between interpolated samples, and window size of 30. After interpolation, the resulting data is re-normalized to represent relative abundances. The resulting trajectory has evenly spaced samples at the desired density, with the values of the features computed as a weighted sum of the original samples as described above. To get similar time points between interpolated trajectories, the first interpolated day for each trajectory was set to be the first multiple of 30 larger than the earliest sample in the original trajectory. When using the exact time points of the data were critical, e.g., when carrying out age prediction tasks, we used the original, non-interpolated data.

### Temporal Alignment

Consider *X* = (*x*_1_…*x*_*n*_) and *Y* = (*y*_1_…*y*_*m*_), two time-series of observations of size n and m, respectively, and some dissimilarity function *d*(*x*_*i*_,*y*_*j*_)≥0 that is defined for every pair of elements *x*_*i*_,*y*_*j*_,*i* ∈ [1,*n*],*j* ∈ [1,*m*]. Importantly, since the alignment algorithm gets as input solely the pairwise dissimilarity matrix, it can be used with a large variety of dissimilarity measures suitable for microbiome data such as Bray-Curtis (as we did in this work) or UniFrac dissimilarities.

Given this input, the DTW algorithm aims to find a *warping function* of length T, denoted: ϕ(ϕ_*x*,*k*_,ϕ_*y*,*k*_),*k* ∈ [1,*T*], while ϕ_*x*,*k*_ ∈ [1,*n*] and ϕ_*y*,*k*_ ∈ [1,*m*]. Simply put, the warping function consists of T pairs of matched indices of elements from the two input time-series. To avoid meaningless loops in the alignment, the resulting series of indices, ϕ_*y*_ and ϕ_*x*_, must be weakly monotonously increasing, such that ϕ_*x*,*i*_≥ϕ_*x*,*i* + 1_ for every i (and similarly for ϕ_*y*_). Moreover, there is often a constraint regarding the matching of the start and end point of the warping functions (see also below), such that ϕ_*x*,1_=ϕ_*y*,1_=1and ϕ_*x*,*T*_=*n*,ϕ_*y*,*T*_=*m*. For each *k* ∈ [1,*T*], the warping function induces a match between samples of the original time series: *x*_ϕ*x*,*k*_is matched to *y*_*ϕy,k*_ (see Figure 1).

Given this wrapping function, the accumulated dissimilarity between the matched samples can now also be calculated as $D_{\phi }\,\left(X,Y\right)=\sum_{k=1}^{T}d\,\left(x_{\phi_{x,k}},y_{\phi_{y,k}}\right)\,w_{k}$. Importantly, in the formula above, *w_k_* is a weight assigned to each pair according to a pre-defined logic referred to as the *step pattern*. The step pattern defines the allowed transitions between consecutive elements in the alignment and their corresponding weights. Unless noted otherwise, we applied in this work the widely used *symmetric2* pattern that allows the following transitions: if the warping function matched indices *i* and *j*, the next match is limited to the following options: (*i* + 1,*j*),(*i*,*j* + 1),(i+1,j+1), with the corresponding weights: (1,1,2). Furthermore, for the accumulated dissimilarities described above to be comparable to each other, it is common to normalize the accumulated dissimilarity using a *normalization factor N*_ϕ_, unique to each step pattern. In the case of symmetric2 step pattern, the normalization factor is the sum of length of the participating time-series: *N* = *n* + *m*. Different step patterns and their influence on the performance of DTW in other domains was a subject of discussion in related literature (Sakoe and Chiba, 1978; Myers et al., 1980). The core idea of the DTW algorithm is that the warping function induces an alignment that minimizes the accumulated dissimilarity: *argmin*_ϕ_*D*_ϕ_(*X*,*Y*). Although the search space is large, the optimal warping path for time-series of length N and M in the general case can be found in *O*(*N* + *M*) using dynamic programming, similarly to the Needleman-Wunsch algorithm for global alignment of sequences (Needleman and Wunsch, 1970). Moreover, since the alignment algorithms aren’t necessarily symmetric, it is also useful to define the first trajectory that participates in the alignment as the *Reference* trajectory, and the second trajectory as the *Query* trajectory.

While an alignment respecting the start and end point constraints described above is referred to as a *Global Alignment*, one or both constraints may be relaxed, and the result is generally referred to as a *Local Alignment*. Global alignments can be used, for example, to assess the overall similarity between two processes or to characterize differences in the temporal dynamics across the two trajectories, while local alignments can be useful for finding regions of notable similarity. An example for the calculation of the optimal global and local alignment on real microbiome data can be seen in Figures 1D,E. There are various sub-types of local alignments, depending on whether the start and/or end point constraints are relaxed for either one or both trajectories. In this work, we limited the scope of local alignment to *open-begin-end* alignments with respect to the query trajectory only, i.e., the reference trajectory can have un-aligned “head” and “tail”, while the query trajectory must be matched in full. The calculation of alignments in this work was carried out using the dtw-python code library (Giorgino, 2009; Tormene et al., 2009).

## Results

As discussed above, the output of the alignment algorithm consists of two main components. The first is the normalized accumulated dissimilarity along the matched samples. This alignment score can essentially be used as a measure for the inherent dissimilarity between two trajectories (i.e., with low scores denoting trajectories that are generally more similar). As such, it can be used as an input to downstream analyses such as clustering, classification, or other statistical comparisons involving dissimilarity measures. Second, the warping curve itself provides information about the mutual relationship between the temporal dynamics of the aligned trajectories (see Figures 1C–E). For instance, if samples that make up a large segment of trajectory A are matched to a small segment in trajectory B, it might suggest that trajectory A has slower dynamics or that it’s somehow delayed in this segment compared to trajectory B. Below, we demonstrate the use of these two components of the alignment algorithm’s output, and how each can contribute to microbiome analyses.

### Using Alignment-Based Scores as a Similarly Measure Between Trajectories

**FIGURE 2:**
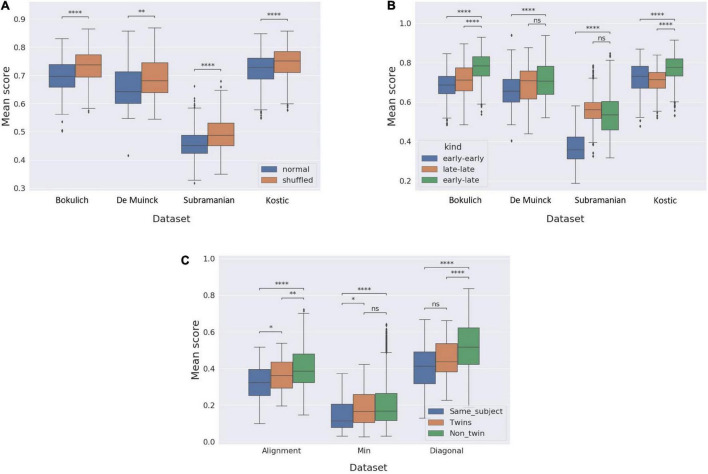
Alignment score as an informative similarity measure. **(A)** Box plot comparing the alignment scores of all pairs of subjects in each dataset to the alignment scores of all pairs of subjects after shuffling the order of samples. **(B)** Box plot comparing the alignment scores of early halves of trajectories aligned to each other (“early-early”), late halves aligned to each other (“late-late”), and early halves aligned to late halves (“early-late”) in each of the datasets. **(C)** Box plot comparing the alignment scores of all pairs of subjects originating in the same trajectory to the alignment scores of trajectories of pairs of twins and the alignment scores of non-related individuals, in the Subramanian dataset. ns: *p* > 0.05; *p ≤ 0.05; ^**^p ≤ 0.01; ^***^p ≤ 0.001; ^*⁣*⁣**^p < 0.0001.

To demonstrate the ability of alignment-based methods to match similar samples, and in turn support temporal analysis of microbiome data, we carried out a series of computational analyses involving the use of the alignment algorithm in a variety of settings. First, we examined how global alignment impacts the similarity measured between a pair of subject trajectories, compared to a naïve similarity score calculated as the mean similarity between the chronologically parallel samples in the two trajectories (i.e., along the diagonal in Figure 1C). For each pair of subjects we trimmed the interpolated trajectories to include only the time points covered by both trajectories. Since global-alignment, by definition, is expected to increase the measured similarity (i.e., minimize dissimilarity), we specifically compared the magnitude of this increase in similarity when applied to real trajectories to the increase in similarity when aligning randomly generated trajectories (using several different settings). As expected, both for the original trajectories and for randomly generated trajectories, global alignment increases similarity compared to the naïve “diagonal” similarity measure, yet, the magnitude of this effect is indeed higher for real trajectories, suggesting that the alignment algorithm captures some underlying temporal dynamics in these real trajectories (Supplementary Figure 1). Next, we compared the global alignment scores for each pair of subjects in each dataset to global alignment scores calculated after shuffling the temporal order of each trajectory. Since the alignment algorithm is only able to match monotonously increasing indices, we hypothesized that alignment scores obtained for the original data will be better than those obtained for shuffled trajectories. Indeed, in all four datasets, the alignment scores for the original data were significantly better (lower dissimilarities) than those calculated for the shuffled data, with Mann-Whitney test *p*-values ranging from 0.01 for the De Muinck dataset to 10^–21^ for the Subramanian dataset (Figure 2A). To further test whether the alignment scores reflect basic biological similarity, we further split each trajectory in all datasets into two halves: an “early-half” and a “late-half.” We then calculated the global alignment scores of the early halves to each other, the early halves to the late halves, and the late halves to each other, for every pair of subjects within each dataset. We hypothesized that since infant microbiome development potentially follows some basic dynamic pattern (that is likely different in early vs. late stages), matching similar halves (“early-early” and “late-late” alignments) will yield better alignment scores than the “early-late” alignments. As expected, indeed in most comparisons made (6 of 8), the early-early and late-late alignments yielded significantly better alignment scores than the early-late alignments (Figure 2B).

Having confirmed that microbiome temporal alignment can distinguish similar trajectories from shuffled or mismatched trajectories, we turned to examine whether the alignment score can also reveal more complex and biologically relevant similarities. Specifically, we hypothesized that alignment may allow us to pinpoint trajectories originating from the same subject vs. those originating from two different subject, and that it can further identify family kinship, yielding, for example, better alignment for siblings than for unrelated subjects. To test this hypothesis, we investigated the Subramanian dataset that includes several pairs of twins. First, we confirmed that microbiome alignment can identify trajectories from the same subject. To this end, we artificially created two different trajectories originating from each subject by splitting each original trajectory (i.e., before interpolation) in the dataset alternately into two trajectories: one including only the even-indexed samples, and the other only the odd-indexed samples. We then interpolated each of these “sub” trajectories (as described above), using window-size of 30 to create a smoother and more even sampling. Since the twin cohort data only contains samples from the first year of life, we cropped all the trajectories to contain only samples from day 0 to 365. We then compared the global alignment scores within three groups: trajectories originating from the same subject, trajectories from pairs of twins, and trajectories from non-related subjects. To benchmark the performance of the alignment method, we also implemented two, more naïve methods for comparing microbiome trajectories: the first, calculating the minimum dissimilarity between the two trajectories (i.e., the minimal dissimilarity score calculated between a sample in the first trajectory to a sample in the second trajectory), and the second, calculating the mean dissimilarity between the chronologically parallel samples in the two trajectories (as also described above). We found that while the alignment-based method yielded significantly better dissimilarity scores for same-subject trajectories than for twins (*p*-value = 0.023), and better dissimilarity for twins than for non-related individuals (*p*-value = 0.006), each of the more naïve methods failed in one of the comparisons (Figure 2C), suggesting that the alignment scores reflect the temporal aspects of the biological similarities and differences resulting from family kinship better than the naïve methods.

Finally, we set out to test whether alignment-based dissimilarities can be linked to more complex biological phenotypes. Specifically, we used the Bokulich data, for which information about diet (breastfed vs. formula; *n* = 31 and 12, respectively) and mode of delivery (vaginally vs. cesarean section; *n* = 24 and 19, respectively) was available. We examined how well alignment scores can partition infant microbiome trajectories based on these phenotypes [using a PERMANOVA (Silverman et al., 2018) test], again, in comparison to more naïve alternative dissimilarity measures. Given the complexity of this task, in this analysis we examined the performance of the alignment algorithm using both the basic symmetric step pattern used above (“*symmetric2*”; see section “Methods”), as well as several other step-patterns previously shown to be useful for tasks in other domains. We specifically considered the three symmetric step patterns described in Sakoe and Chiba (1978) (denoted *symmetricP05*, *symmetricP01*, and *symmetricP2*), and the step pattern from Myers et al. (1980) (denoted *TypeIIIc*). Notably, the selection of a step-pattern to a specific domain or task is challenging to determine a-priori, and is usually done empirically through experiments. With that in mind, in the following analysis we did not aim to test whether alignment-based dissimilarities are better than naïve methods for partitioning different biological phenotypes, but rather to explore the performance of DTW with different step-patterns and how this may relate to different biological phenotypes. Indeed, our analysis suggested that alignment-based dissimilarity metrics could exhibit beneficial performances for both phenotypes, albeit different step-patterns performed better in each scenario, confirming that the specific step function used can impact the obtained alignment and that different biological phenotypes may be more or less suitable for a given step function. Specifically, for the diet phenotype, the typeIIIc alignment score yielded a marginally significant *p*-value of 0.052, while the minimal *p*-value in any of the alternative methods (obtained by the min method) was 0.071 (symmetric2, symmetricP2, symmetricP01, and symmetricP05 resulted in *p*-values of 0.183, 0.083, 0.075, and 0.09, respectively, and the mean and diagonal methods resulted in *p*-values of 0.102 and 0.106). For the mode of delivery phenotype, all methods yielded significant *p*-values, with the lowest *p*-value of 0.002 obtained by the symmetricP05 method, while the lowest *p*-value obtained by a non-alignment method was 0.006 (obtained again by the min method; symmetric2, symmetricP2, symmetricP01, and typeIIIc resulted in *p*-values of 0.005, 0.006, 0.007, and 0.009, respectively, and the mean and diagonal methods resulted in *p*-values of 0.011 and 0.012). Given these findings, the use of temporal alignment in future microbiome studies should likely be accompanied by empirical test of different similarity measures and specifically should take different DTW-based approaches into account.

### Using Alignment-Based Matching for Inferring Microbiome Dynamics

Below, we set out to show that not only the alignment score, but also the optimal pairwise matching of samples obtained by the alignment algorithm carry meaningful and useful information concerning the dynamics of the microbiome. To confirm this, we implemented a pipeline that uses local alignment for predicting the chronological age of the individual from whom samples were obtained based on its microbiome composition. Using the microbiome to predict a subject’s age was first demonstrated by Subramanian et al. (2014), using a random forest regression for this task and utilizing the predictions to assess the relative maturity of the microbiome of children. Since the focus of our work is to examine the abilities and properties of alignment-based methods in different settings (rather than to improve age prediction accuracy), we did not benchmark the performance of the alignment-based age prediction pipeline against such machine learning based methods, but rather verified that the alignment-based approach successfully predicts ages based on the microbiome and identifies similar patterns to those observed using machine learning methods.

**FIGURE 3:**
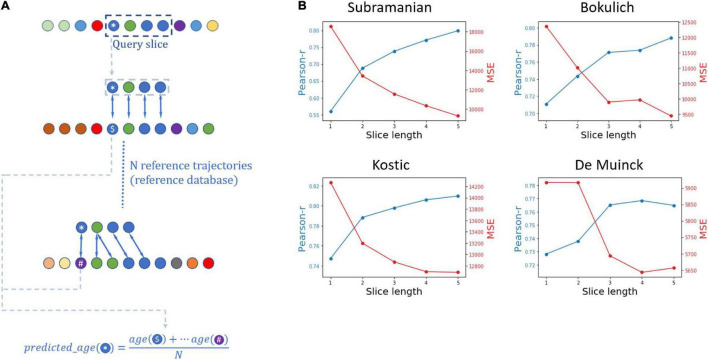
Using microbiome temporal alignment for age prediction. **(A)** A schematic illustration of the age prediction framework for a 4-samples-long slice. The query in the age prediction framework is a slice from a trajectory, marked by the dashed box. The ages of samples in the query are assumed to be unknown, and for simplicity purposes the age prediction framework aims to predict the age of the first sample of the slice, marked by an asterisk. The local alignment between the query slice and each trajectory from a reference dataset is calculated, and the predicted age is then calculated as the mean age of the samples to which the query sample was aligned. **(B)** Results of the simulations evaluating the performance of alignment-based age prediction. Shown are the Pearson’s *r* values for the correlation between the predicted and real ages, alongside mean square error (MSE) values, for each slice length in the four datasets analyzed in our study.

A schematic illustration of the age prediction framework is presented in Figure 3A. The input to the prediction pipeline is a continuous segment (which we term here “slice”) of a given trajectory and a collection of reference trajectories. We also assume that the chronological ages of the samples in the slice are unknown, but that the age of each sample in each of the reference trajectories are provided. Given this input, our pipeline aims to predict the age of the first sample (for simplicity) of the input slice by local aligning the slice to each of the reference trajectories in the collection and calculating the mean age of all the samples in the reference trajectories to which the first sample in the slice was matched. Since these analyses focus on predicting the chronological age of microbiome samples, we carried out all the experiments described below using the original non-interpolated version of the datasets.

To evaluate the performance of this age prediction approach we randomly chose an individual from a given dataset, randomly cropped a continuous 5-sample slice from that individual’s trajectory, aligned the resulting slice against all other trajectories in the dataset (except the trajectory from which the slice was taken), and calculated the predicted age of the first sample in the slice as described above. We repeated this procedure 300 times, in each of the four datasets. Moreover, to assess the information capture by the alignment method and the impact of the slice length, we repeated this prediction not only for the full 5-sample slice but also for prefixes of each slice at varying length (from 1 to 5). The prediction accuracy was then quantified using the Pearson’s *r* and mean square error (MSE) between the predicted age and the real age for each dataset and for each slice length (Figure 3B). Indeed, this analysis has demonstrated that in all datasets and across all slice lengths (from 1 to 5), the correlation between the real and predicted age was statistically significant, suggesting that the alignment method is effective in matching similar temporal patterns. Furthermore, using longer slices resulted in a more accurate age prediction (both in terms of the correlations between predicted and real age and in terms of the mean square error of the prediction), indicating that the alignment algorithm successfully utilizes continual temporal trends beyond the naïve match with the closest sample.

While the results reported above clearly demonstrate the ability of the temporal alignment-based approach to support age prediction by utilizing temporal patterns in longitudinal microbiome data of healthy subjects, we next set out to examine whether it can similarly serve to quantify potential delay in microbiome development [as previously done by machine learning (Subramanian et al., 2014)] that may result from, for example, malnutrition. To this end, we applied our age prediction pipeline to the Subramanian dataset described above. Notably, this dataset contains microbiome data sampled from Bangladeshi children with severe acute malnutrition (SAM) who were admitted to a hospital for treatment. The children were sampled at admittance, before being treated (“Acute phase” samples), during treatment (“Treatment” samples), and several times during follow up.

As noted, the original paper describing this cohort (Subramanian et al., 2014) utilized machine learning models trained on a healthy control cohort to predict the ages of the malnourished children during the different experimental phases and calculated the difference between the predicted and chronological ages, referred to as “relative microbiota maturity.” Negative microbiota maturity values (i.e., the infant’s microbiome resembles the microbiome of a younger child) suggest a lag in the microbial community’s development. The researchers observed that compared to healthy children, malnourished kids had a significantly immature microbiome. While they didn’t witness a significant effect in the treatment phase, they did observe a significant improvement starting from the first follow-up sample, which regressed back to an immaturity index similar to the intake time after 4 months of follow up.

**FIGURE 4:**
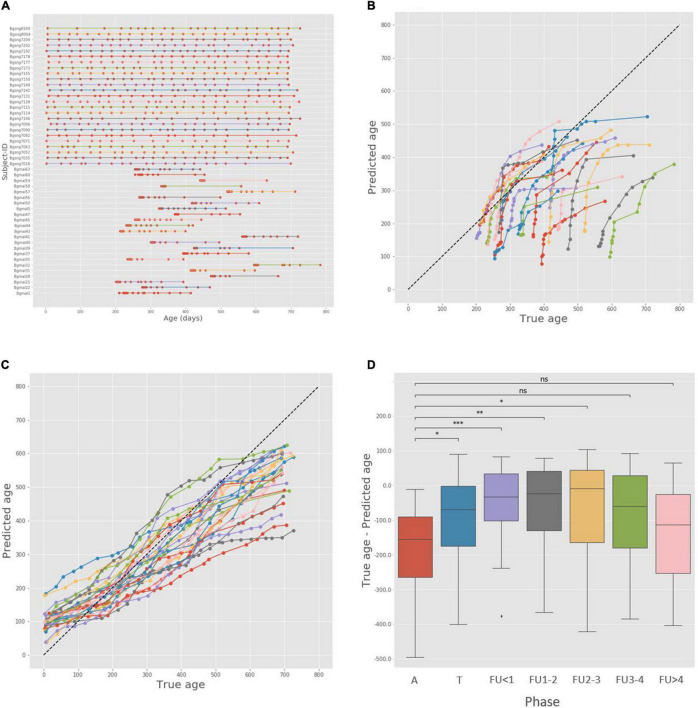
Alignment-based age analysis and maturity indices in the Bangladesh cohort. **(A)** Ages of the malnourished and healthy children participating in the analysis. Subject-IDs of healthy children start with the prefix Bgsng (Bangladeshi singleton, found in the top half of the figure), and Subject-IDs of malnourished children start with the prefix Bgmal, found in the bottom half of the figure. **(B)** True and predicted age for all severe acute malnutrition (SAM) trajectories. The points representing each subject are colored similarly and connected with a line. **(C)** True and predicted age for all healthy singleton trajectories. **(D)** Box plot of relative maturity indices, calculated as the difference between true age and predicted age, across the different phase groups. Since each subject has multiple samples from each phase, we only used the latest sample in each phase per subject. A – Acute phase; T – Treatment; FU < 1 – Follow-up, under 1 month; FU1-2 – Follow-up, 1 to 2 months; FU2-3 – Follow-up, 2 to 3 months; FU3-4 – Follow-up, 3 to 4 months; FU > 4 – Follow-up, over 4 months. ns: *p* > 0.05; *p ≤ 0.05; ^**^p ≤ 0.01; ^***^p ≤ 0.001; ^*⁣*⁣**^p < 0.0001.

To examine whether an alignment-based method can be used as an alternative approach for such an analysis, we used the healthy singleton infants (i.e., without the healthy twins and triplets’ data) as a healthy reference group (Figure 4A), as done in the original study. We then used our age prediction pipeline to predict the age of each sample in the SAM infants’ trajectories by local-aligning it to each of the healthy-reference infants, and predicting the age as above by calculating the mean age of all reference samples matched to each sample of a SAM infant. Unlike in the previous age-prediction task, this time we predicted the ages of all samples in each trajectory rather than only the first sample. As control, we carried out a similar analysis but using healthy individuals as the subjects of age prediction. Specifically, in this analysis, the prediction pipeline described above was applied to the healthy individuals dataset with a leave-one-out approach (i.e., in each iteration, one healthy individual was selected at random, training the age-prediction model on all other healthy individuals and using the obtained model to predict the age of samples from this left-out individual).

The true and predicted ages of all SAM trajectories are shown in Figure 4B. A qualitative examination of the results suggests similar trends to those reported in the original study; first, the majority of samples are found under the diagonal, meaning that the predicted ages are generally smaller than the true ages and exhibit microbiome immaturity. Furthermore, all subjects follow a similar dynamic scheme – a phase of moving toward the diagonal, in agreement with previous findings that the immature microbiome is able to “catch up” with the healthy reference following treatment, and later a phase of moving away from the diagonal, suggesting a regression – a period of stagnated development and a drop back to lower maturity. Importantly, such a behavior was not observed in the healthy control group (Figure 4C). Instead, in these healthy individuals, predicted ages tended to be markedly more aligned along the diagonal (i.e., predicted age was generally similar to chronological age) and did not exhibit the distinct curvature observed in the malnourished individuals but rather a more monotonic pattern of maturation. We further examined the average maturity indices of samples in the different phases of the experiment, again finding that they mirror the observations above (Figure 4D). A significant improvement in maturity relative to the acute phase is already witnessed in the treatment phase and remains significant until the follow-up period 3–4 month after treatment, when it regresses back to a statistically similar score to the acute phase. Combined, these observations suggest that the alignment method is effective in uncovering temporal dynamics in a complex setting of malnutrition as well as in the healthy settings presented above.

## Discussion

In this work, we introduced the concept of temporal alignment of microbiome data and explored its importance for longitudinal analysis of microbiome data. The underlying assumption of this work is that differences in the pace and dynamics of microbiome-based temporal processes can often overshadow similarities and differences between such processes, and that alignment-based methods can account for these shifts, facilitating the extraction of information that may be otherwise masked. We focused specifically on the process of microbiome development during the first years of life as a model for highly dynamic and complex microbiome process and explored the application of alignment-based methods on several datasets of infant microbiomes with various demographic and medical characteristics, and preprocessed using various protocols.

To validate the outcomes of the alignment framework, and specifically the usefulness of alignment-based similarity measure (i.e., the obtained alignment score), we showed that the alignment score can distinguish similar trajectories from artificially shuffled or mismatched trajectories, and that it outperforms more naïve methods as a measure of similarity between trajectories. Importantly, however, while in the potentially simpler task of inferring family kinship, the “out of the box” alignment algorithm was sufficient to outperform other benchmark methods, more complex phenotypes (such as diet or mode of delivery) required more sophisticated configurations with different step patterns. In addition to the use of the alignment score, we also showed that the obtained alignment curve (including the resulting pairwise mapping of samples), can reveal valuable insights regarding temporal dynamics in the data. While we demonstrated this concept using the age prediction framework and the alignments of malnourished to healthy Bangladeshi kids, analyses in the same vein can be carried out on diverse data sources.

The recognition that the temporal behavior of microbial communities may have important impact on the host’s health is continuously promoting microbiome studies focusing on longitudinal data. Yet, in order to benefit from such data and to clearly characterize their association with various biological phenotypes, methodologies that take temporal aspects into account are necessary. Specifically, since the comparison of microbiomes across different individuals is a fundamental step in revealing such associations, a framework for temporally informed comparisons that considers not only the microbiome composition at a given time point, but also temporal patterns, is crucial. Importantly, however, while we believe that our work clearly demonstrated the potential of using alignment scores as a measure of similarity between trajectories, further research regarding the performance of the alignment method, both in various experimental settings and using different configurations, is still required to unlock the full potential of this framework. Among the aspects that can be examined is the use of various other local and global constraints in the alignment process and/or the step-patterns used in this work, many of which are widely discussed in the literature by researchers in other domains. It is also worth noting that while the process of microbiome maturation (which is characterized by distinct and substantial temporal dynamics) is a fertile setting for exploring and benefiting from temporal alignment methods, such methods might be less beneficial in the context of healthy adult gut microbiomes, which were previously shown to have a relatively stable and stationary nature (Mehta et al., 2018). Moreover, in some cases, differences in pace and dynamic are not an artifact interfering with similarity measures but rather a clinically significant feature (e.g., as seen in the malnourished children or potentially in fast vs. slow disease onset). In such cases, using alignment to measure similarly may mask such biologically relevant trends and should be applied with caution.

It is also important to note that although the collection of longitudinal microbiome data is becoming more common, there is a built-in tradeoff when designing a longitudinal study between the period covered by the experiment, the density of sample collection, and the total number of participating subjects. Focusing on any of these aspects at the expense of others poses different challenges when analyzing the data. For example, dense sampling might uncover temporal dynamics that may be otherwise hidden (De Muinck and Trosvik, 2018), but might be achieved at the expense of the number of subjects, resulting in lower statistical power. With that in mind, cohorts that cover a significant period of time, and include both a large number of subjects and a dense temporal sampling, are still scarce. The collection of such data is of great importance for further understanding of the temporal dynamics of the microbiome, as well as for developing temporal analysis methodologies. Moreover, interestingly, the four datasets used in our study were subject to somewhat different preprocessing protocols (including, for example, both OTU-based and ASV-based data), suggesting that reported findings are potentially robust to feature table generation techniques. Yet, the collection of additional longitudinal datasets and their processing with a variety of methods could further characterize the impact of longitudinal data processing on downstream analyses and specifically on temporal alignment.

To conclude, we believe that the collection and analysis of longitudinal data is one of the important next frontiers in microbiome research. Based on the work presented here, using the development of the microbiome as a case study for highly dynamic longitudinal microbiome data, we showed that temporal alignment-based methods might contribute greatly to the understanding of the temporal dynamics in the developing microbiome, and in turn, the interplay between the microbiome and various medical and biological conditions.

## Data Availability Statement

All the raw data used for the analysis is available online, as described in the section “Data Acquisition and Pre-processing” segment. The code used for applying the temporal alignment approach, as well as the code notebooks used to generate the various results and figures that appear in this manuscript are available on https://github.com/borenstein-lab/microbiome_temporal_alignment.

## Author Contributions

RA and EB designed the research, interpreted the results, and wrote the manuscript. RA obtained and processed the datasets and performed all computational analyses. Both authors read and approved the final manuscript.

## Conflict of Interest

The authors declare that the research was conducted in the absence of any commercial or financial relationships that could be construed as a potential conflict of interest.

## Publisher’s Note

All claims expressed in this article are solely those of the authors and do not necessarily represent those of their affiliated organizations, or those of the publisher, the editors and the reviewers. Any product that may be evaluated in this article, or claim that may be made by its manufacturer, is not guaranteed or endorsed by the publisher.

## Footnotes

1
https://diabimmune.broadinstitute.org/diabimmune

2
https://gordonlab.wustl.edu/
