# Supplementary material for: Temporal Alignment of Longitudinal Microbiome Data
Source: Front Microbiol. 2022 Jun 22;13:909313. doi: 10.3389/fmicb.2022.909313 (PMC9257075; doi:10.3389/fmicb.2022.909313)
Supplement: Supplementary file 1 [file Image_1.PDF]

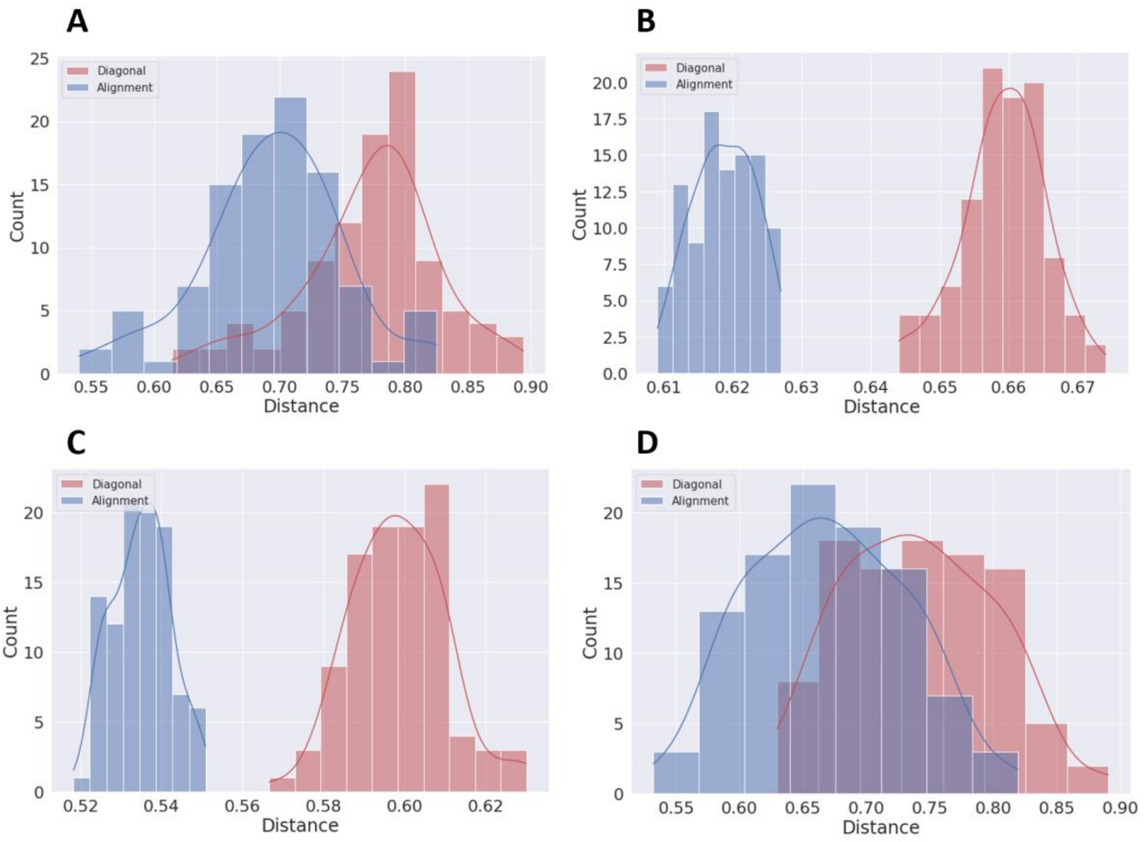

Supplementary Figure 1 – The impact of global alignment on pairwise similarity between trajectories in real vs. randomly generated data. Histograms and Kernel Density Estimates (KDE) of calculated distances between 100 randomly selected pairs of trajectories using global alignment (blue) and a naïve 'diagonal' similarity (calculated as the mean similarity between chronologically parallel samples in the two trajectories; red), for **(A)** real microbiome trajectories from the DIABIMMUNE dataset, and for **(B-D)** three randomly generated datasets, each with the same number of individuals and the same number of samples per individual as in the real data. The randomly generated datasets include: (B) A simulated dataset of trajectories where all feature values were sampled from a Gaussian distribution with mean and STD as calculated across all features and all individuals in the original dataset; (C) A simulated dataset of trajectories where each feature values were sampled from a Gaussian distribution with mean and STD as calculated for the corresponding feature (i.e., taxon) across all individuals in the original dataset; (D) A simulated dataset of trajectories where each feature values in each individual were sampled from a Gaussian distribution with mean and STD as calculated for the corresponding feature and the corresponding individual in the original dataset. As seen in the figure, global alignment, by definition, results in increased similarities (lower distances) even in simulated trajectories, yet the magnitude of this effect (i.e., by how much similarity increases) is more pronounced in real data trajectories (note the different scale of the X-axis). Specifically, the difference in means in the 4 panels is (A) 0.078, (B) 0.041, (C) 0.064, and (D) 0.072.
